# Exposure to positively- and negatively-charged plasma cluster ions impairs IgE-binding capacity of indoor cat and fungal allergens

**DOI:** 10.1186/s40413-016-0118-z

**Published:** 2016-09-06

**Authors:** Kazuo Nishikawa, Takashi Fujimura, Yasuhiro Ota, Takuya Abe, Kareem Gamal ElRamlawy, Miyako Nakano, Tomoaki Takado, Akira Uenishi, Hidechika Kawazoe, Yoshinori Sekoguchi, Akihiko Tanaka, Kazuhisa Ono, Seiji Kawamoto

**Affiliations:** 1 Hiroshima Research Center for Healthy Aging (HiHA), Department of Molecular Biotechnology, Graduate School of Advanced Sciences of Matter, Hiroshima University, 1-3-1 Kagamiyama, Higashi-Hiroshima, Hiroshima 730-8530 Japan; 2 Engineering Department III, Plasma cluster Equipment Division, Health and Environment Systems Group, SHARP Corporation, Osaka, Japan; 3 Division of Allergology and Respiratory Medicine, Department of Medicine, Showa University, Tokyo, Japan; 4 Department of Food Sciences and Biotechnology, Faculty of Life Sciences, Hiroshima Institute of Technology, Hiroshima, Japan

**Keywords:** Airborne allergen, Asp f 1, Asthma, Cat allergen, Environmental control, Fel d 1, Fungal allergen, Immunoglobulin E, Positively- and negatively-charged cluster ions

## Abstract

**Background:**

Environmental control to reduce the amount of allergens in a living place is thought to be important to avoid sensitization to airborne allergens. However, efficacy of environmental control on inactivation of airborne allergens is not fully investigated. We have previously reported that positively- and negatively-charged plasma cluster ions (PC-ions) reduce the IgE-binding capacity of crude allergens from Japanese cedar pollen as important seasonal airborne allergens. Cat (*Felis domesticus*) and fungus (*Aspergillus fumigatus*) are also important sources of common airborne allergens in living spaces throughout the year, and early sensitization with those allergens is considered to be a risk factor for future development of allergic rhinitis, pollinosis and asthma. The aim of this study is to examine whether the PC-ions reduce the IgE-binding capacity of a cat major allergen (Fel d 1) and fungal allergens in an experimental condition.

**Methods:**

Fel d 1, crude fungal extract, or a fungal major allergen Asp f 1, was treated with PC-ions for 6 h in an experimental cylindrical apparatus. Sham-treated allergens were prepared in the same experimental apparatus without generation of PC-ions. The degradation of the PC-ions-treated Fel d 1 was analyzed by SDS-PAGE, and the IgE-binding capacity of the PC-ions-treated allergens was analyzed by ELISA inhibition assay.

**Results:**

Exposure of Fel d 1, crude fungal extract and Asp f 1 to PC-ions significantly decreased protein content of Fel d 1 or Asp f 1, respectively. SDS-PAGE analysis suggested that the decreased Fel d 1 content upon exposure with PC-ions was attributable to protein degradation. ELISA inhibition indicated that the PC-ions treatment significantly impaired IgE-binding capacities of Fel d 1, crude fungal allergens, and Asp f 1 compared to sham treatment.

**Discussion:**

Our data suggest that treatment with PC-ions not only reduce indoor cat and fungal allergens, but also impair their allergenicity.

**Conclusion:**

These results suggest that environmental control with PC-ions is useful for inactivation of indoor cat and fungal allergens.

## Background

High amounts of airborne allergens that can be found in living spaces can be a risk factor for sensitization to allergens and elicitation of allergic symptoms, such as rhinitis and asthmatic attacks. Environmental control to clean air pollutants can lead to the amelioration of allergic symptoms. Moreover, environmental control using an air filtration unit resulted in the significant reduction of the quantity of inhaled airborne allergens in a room. Such reductions in the amounts of airborne allergen may result in positive impacts that can improve clinical symptoms [1, 2]. We previously reported that environmental control using an air cleaner with positively- and negatively-charged plasma cluster ions (PC-ions) induced protein degradation and inactivation of atomized Japanese cedar (*Cryptomeria japonica*) pollen allergens [3]. Therefore, we hypothesized that PC-ions might also degrade and inactivate common airborne allergens. In this study, we focused on cat and fungal allergens as common indoor airborne allergens, because cats and fungal allergens represent a significant risk factor to induce respiratory disorders including asthmatic attacks throughout the year [4, 5]. It is more important to investigate PC-ions can inactivate perennial airborne allergens than seasonal allergens for allergic patients. Therefore, we analyzed whether the PC-ions can impair IgE-binding capacity of a cat major allergen, Fel d 1, and fungal allergens as important environmental airborne allergens to induce allergic rhinitis and asthma attacks throughout the year.

Cat (*Felis domesticus*) allergens represent some of the most important airborne allergens that can induce severe allergic rhinitis and asthma. Sensitization to cat allergens in childhood is thought to be a risk factor for the future development of pollinosis, asthma, and allergic eczema [4, 6]. In Japan, a survey of 504 asthma patients conducted in 1993–1994 reported that 70 % of 44 patients who kept cats and 34 % of 394 patients who had never kept cats were positive for cat-specific IgE, as indicated by CAP-RAST score ≥2 [7]. Notably, individuals who never had a cat can also be sensitized to airborne cat allergens as a consequence of exposure to airborne cat allergens outdoors and in public institutions. Cat allergens have been detected in school classrooms in the absence of cats; furthermore, indoor airborne pollutants are more toxic than those that are found outdoors, and these could induce inflammatory and allergic reactions [8]. Airborne cat allergens are thought to be transferred and deposited indoors when attached on the clothes or hair of cat owners [9]. Therefore, sensitization to cat allergens can occur in individuals who do not keep a cat.

Feline uteroglobin was identified as a major allergen for cat allergy and was termed Fel d 1 [10]. Fel d 1 is a tetrameric glycoprotein that is formed by two heterodimers and has an apparent ~38 kDa molecular weight. The heterodimer is composed of two separate chains with apparent molecular weight of 4–5 and 14–20 kDa under denatured condition, as chain 1 and chain 2, respectively [11, 12]. Immunoblotting analysis showed both chain 1 and chain 2 had IgE-binding capacity with cat-allergic patients’ sera and the IgE-binding frequency was 100 % (6/6) and 33 % (2/6) for the chain 1 and chain 2, respectively, in six cat-allergic patients’ IgE. The chain 1 and chain 2 were dissociated in native Fel d 1 after denaturation by such high pH condition or reduced condition with SDS [12]. IgE titers specific to Fel d 1 in cat-sensitized asthma patients were significantly higher than those in cat-sensitized rhinitis patients. Therefore, elevated titers of Fel d 1-specific IgE are thought to be a risk factor for asthma in children who are allergic to cats [13]. These reports showed the immunological importance of Fel d 1 as a major allergen related to cat allergy.

Allergenic fungus (*Aspergillus fumigatus*) is also an important source of airborne allergens and has been a major course of allergic bronchopulmonary aspergillosis. Twenty three allergens from *A. fumigatus* have registered on the official website for the systematic allergen nomenclature that is approved by the World Health Organization and International Union of Immunological Societies (WHO/IUIS) Allergen Nomenclature Sub-committee. Asp f 1 and Asp f 2 are reported to be major allergens for *A. fumigatus* among the registered allergens [14]. Asp f 1 has identified as an IgE-binding protein belonged to mitogillin family of cytotoxins from *A. fumigatus*. Asp f 1 is an 18 kDa protein with 85 % (11/13) IgE-binding frequency within asthma or allergic bronchopulmonary aspergillosis patients [15]. Airborne fungal allergen exposures increase degree of atopy and visit to emergency department or urgent care for asthma [5]. Environmental control including decrease of airborne fungal allergens is considered to be important to reduce the incidence of asthma attacks for both childhood and adult asthma.

Herein, we showed the degradation and impairment of IgE-binding capacity of Fel d 1, crude fungal allergen, and Asp f 1 by PC-ions in experimental cylindrical condition.

## Methods

### Reagents and plasma

A cat major allergen, Fel d 1, and a fungal major allergen, Asp f 1, were purchased from INDOOR Biotechnologies (Charlottesville, VA, USA). Crude fungal extract (CFE) was purchased from ITEA (Tokyo, Japan). All chemicals were purchased from Katayama Chemical Industries (Osaka, Japan), Sigma-Aldrich (St. Louis, MO, USA) or Nacalai Tesque (Kyoto, Japan) unless otherwise indicated. Plasma was obtained from two cat-allergic asthma patients who exhibited positive CAP-RAST score to Fel d 1 and from three fungi-allergic asthma patients who showed positive CAP-RAST score to Asp f 1. The plasma were stored at −30 °C until use. All studies that used human samples were approved by the institutional ethics committee at Showa University School of Medicine (Tokyo, Japan).

### Treatment of positively- and negatively-charged plasma cluster ions

**Fig. 1:**
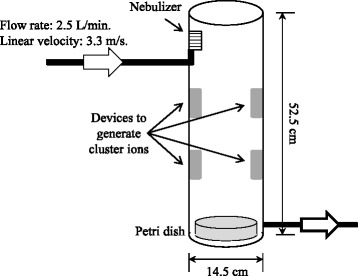
Cylindrical apparatus for PC-ions exposure. PC-ions were generated in four Plasma cluster™ devices (*shown as gray boxes*) that were placed inside of wall of the apparatus. PC-ions were emitted from the devices and filled the apparatus at 2.5 × 10^4^ ions/cm^3^. A “mist” solution containing allergen was generated from a nebulizer located on the top of the apparatus, which is shown in a box with horizontal stripe. The solution was shed at 2.5 L/min (flow rate) and 3.3 m/s (linear velocity). A petri dish was placed beneath the apparatus to collect the PC-ions- or sham-treated allergen solution

Airborne allergens; Fel d 1, CFE and Asp f 1, were treated with positively- and negatively-charged plasma cluster ions (PC-ions), as described previously [3]. Briefly, a cylindrical apparatus (14.5 cm diameter × 52.5 cm height) was filled with PC-ions (2.5 × 10^4^ PC-ions/cm^3^) for 6 h, and then 221 mL of 1 mg/ml Fel d 1 solution or 666 ml of 40 ng/ml CFE or Asp f 1 solution was nebulized from top of the apparatus to expose the PC-ions. Nebulized solution was collected in a 10 cm petri dish (BD Biosciences, San Diego, CA, USA) that was placed underneath the apparatus (Fig. 1). Sham-treated allergen was prepared in the same condition, but without generating the PC-ions.

### Quantification of Fel d 1 and Asp f 1 by ELISA and mass spectrometric analysis

Amount of Fel d 1 and Asp f 1 was quantified using a Fel d 1- or Asp f 1-specific enzyme-linked immunosorbent assay (ELISA) kit (INDOOR Biotechnologies) following the manufacturer’s instructions. Prior to quantification of Fel d 1 by ELISA, CFE and Fel d 1 solution after PC-ions or sham treatment were 1,250-fold concentrated by lyophilization. Fel d 1 that was collected after PC-ions or sham treatment was separated by sodium dodecyl sulfate polyacrylamide gel electrophoresis (SDS-PAGE) under reducing condition and proteins were visualized by silver staining following the manufacturer’s instructions (Cosmo Bio, Tokyo, Japan). Proteins separated on SDS-PAGE were identified by liquid chromatography (LC) connected to a mass spectrometer (MS, LTQ Orbitrap XL, Thermo Fisher Scientific, Rockford, IL, USA) using a HPLC column (Develosil ODS-HG-5, Nomura Chemical, Aichi, Japan) and Xcalibur software v2.0.7 (Thermo Fisher Scientific), as described elsewhere [16].

### ELISA inhibition

The immunoglobulin E (IgE)-binding capacity of PC-ions-treated allergens for pooled plasma from patients sensitized with each allergen was analyzed using an ELISA inhibition assay, as described previously with slight modifications [3]. Briefly, a 96-well microtiter plate (NUNC maxisorp, Thermo Fisher scientific) was coated with 0.1 ml of 200 ng/ml sham-treated Fel d 1, CFE or Asp f 1 dissolved in 100 mM bicarbonate buffer (pH 9.4) overnight at 4 °C. After washing three times with phosphate buffered saline containing 0.05 % Tween 20 (PBST), plate were blocked with PBST containing 3 % skim milk and 1 % bovine serum albumin (BSA) for 2 h. Pooled plasma was diluted 80-fold from two Fel d 1-positive asthmatic patients or 12.5-fold from three Asp f 1-positive asthmatic patients, which was preincubated with serial dilutions of PC-ions- or sham-treated Fel d 1, CFE or Asp f 1 overnight at 4 °C. Then, pooled plasma was applied to sham-treated Fel d 1-, CFE- or Asp f 1-coated plates, which were incubated for 12 h at 4 °C. After washing with PBST three times, plates were incubated with biotinylated anti-human IgE monoclonal antibody (Vector Laboratories, Burlingame, CA, USA) diluted 1:1,000 for 1 h at room temperature, followed by 1 h incubation with alkaline phosphate-conjugated streptavidin (Jackson ImmunoResearch Laboratories, West Grove, PA, USA). To determine the amount of IgE-binding, Attophos substrate solution (Promega, Fitchburg, WI, USA) was added to the plate and fluorescence intensity was measured using a Wallac 1420 ARVOsx Multilabel Counter (Perkin-Elmer, Downers Grove, IL, USA).

### Statistical analysis

Statistical analyses were performed using non-repeated ANOVA. For differences that were found to be significant, post-hoc analysis using a Student-Newman-Keuls test was performed. A threshold for statistical significance was set at *P* < 0.01.

## Results

### PC-ions decrease protein content of a cat major allergen, Fel d 1

**Fig. 2:**
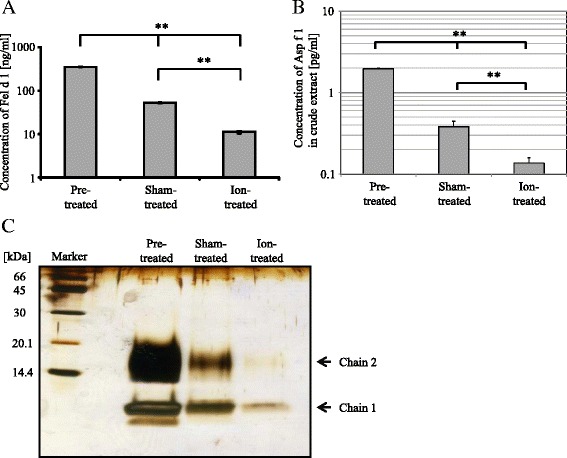
Degradation of Fel d 1 and CFE after PC-ions treatment. Concentrations of Fel d 1 (**a**) or Asp f 1 contained in CFE (**b**) in solution before and after sham or PC-ions treatment, as measured by ELISA, are represented as pre-treated, sham-treated and ion-treated, respectively. Concentrations of allergens are represented as means ± standard deviation. Significant differences at the level of **P < 0.01 was estimated by the post-hoc Student-Newman-Keuls test after non-repeated ANOVA. **c** Solutions of Fel d 1 before treatment and collected in the petri dish after sham or PC-ions treatment were loaded on a SDS-PAGE gel under reducing condition, which were labeled as pre-treated, sham-treated, and ion-treated, respectively. After visualizing bands by silver staining, the amino acid sequences of the main 15.0 and 6.5 kDa bands (chain 2 and chain 1, respectively) were analyzed by LC-MS and were confirmed to match the amino acid sequence of Fel d 1. Chain 1 and chain 2 for Fel d 1 were indicated with arrows. The lane labeled as Marker represents a molecular weight marker

We previously reported that PC-ions inactivated the allergenic activity of crude allergen extracted from Japanese cedar (*Cryptomeria japonica*) pollen [3]. This finding prompted us to investigate whether PC-ions could also inactivate the IgE-binding activity of cat and fungal allergens, as these are among the most important airborne indoor allergens that can induce severe allergic rhinitis and asthma attacks. After treatment with PC-ions, the concentration of Fel d 1 was significantly reduced compared with those measured before treatment and after sham treatment (351.1, 52.6, and 11.4 ng/ml for before treatment, after sham and PC-ions treatments, respectively; Fig. 2a). Concentration of Fel d 1 after sham treatment was also significantly reduced compared with that before treatment. The reduction was thought to be a consequence of non-specific degradation by vaporization of solution in the apparatus (Fig. 1). Indeed, the concentration of Fel d 1 was reduced by 79.3 % after PC-ions treatment compared with that after sham treatment. SDS-PAGE analysis indicated that both the 16.0 kDa (chain 2) and 6.5 kDa (chain 1) proteins were significantly reduced after PC-ions treatment compared with those before treatment and after sham treatment (Fig. 2c). We confirmed that the 16.0 and 6.5 kDa proteins were subunits of Fel d 1 by analyzing of internal amino acid sequences after trypsin digestion using LC-MS (data not shown).

### PC-ions impair the IgE-binding capacity of Fel d 1

**Fig. 3:**
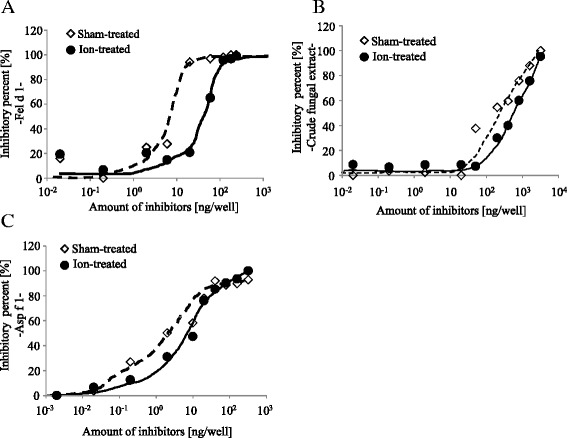
Impairment of IgE-binding capacity of Fel d 1, CFE and Asp f 1 after PC-ions treatment. The IgE-binding capacity of Fel d 1 (**a**), CFE (**b**) or Asp f 1 (**c**) after PC-ions treatment was analyzed by ELISA inhibition assay. Inhibition of binding between a pooled plasma from patients and sham-treated Fel d 1, CFE or Asp f 1 was analyzed using sham- and PC-ions-treated Fel d 1, CFE or Asp f 1, shown as open diamonds with a broken line (sham-treated) and filled circles with a line (ion-treated), respectively

We then analyzed the IgE-binding capacity of Fel d 1 after PC-ions or sham treatment. We tested IgE-binding capacity of PC-ions-treated Fel d 1 by inhibitory activity between sham-treated Fel d 1 and a pooled plasma from Fel d 1-positive patients, as only nebulization could reduce IgE-binding capacity for Fel d 1, as suggested by the protein degradation analysis (Fig. 2a and c). The inhibition curve for Fel d 1 after PC-ions treatment was shifted towards a higher allergen dose compared with that of sham treatment (Fig. 3a). The amount of protein needed achieve 50 % inhibition of binding between sham-treated-Fel d 1 and pooled plasma was 53.5 and 12.4 ng for PC-ions- and sham-treated Fel d 1, respectively. This finding showed 76.9 % of IgE-binding capacity was impaired by PC-ions treatment. These data strongly suggested that PC-ions inactivated indoor cat allergens by degrading Fel d 1.

### PC-ions also impair the IgE-binding capacity of fungal allergens

We also analyzed the impairment of IgE-binding capacity of fungal allergens by PC-ions treatment. The amount of Asp f 1 in CFE after both sham and PC-ions treatments was significantly decreased compared with that before treatment as same as the case of Fel d 1, and the amount of Asp f 1 after PC-ions treatment was significantly reduced compared with that after sham treatment (1.97, 0.38 and 0.14 pg/ml before treatment and after sham or PC-ions treatment, respectively; Fig. 2b). We also nebulized a purified Asp f 1 in the cylindrical apparatus. The amount of the Asp f 1 was also significantly decreased after nebulizing in the apparatus as well as the case of CFE, however, the amount of Asp f 1 after PC-ions treatment was comparable with that after sham treatment (6.1, 1.9 and 1.7 ng/ml before treatment and after sham or PC-ions treatment, respectively; data not shown).

We then analyzed IgE-binding capacity of CFE and Asp f 1after PC-ions treatment. The inhibition curve for PC-ions-treated CFE was shifted to high dose of inhibitors compared to sham-treated CFE (Fig. 3b). The amount of inhibitors to achieve 50 % inhibition of sham-treated CFE and a pooled plasma were 159 ng for sham-treated CFE and 601 ng for PC-ions-treated CFE, respectively (Fig. 3b). The result showed 73.5 % reduction of IgE-binding capacity by PC-ions treatment for CFE. To confirm the inactivation of IgE-binding capacity of Asp f 1 by PC-ions, IgE-binding capacity of Asp f 1 after PC-ions treatment was analyzed. For the inhalation as a purified Asp f 1, the amount of inhibitors to achieve 50 % inhibition of sham- and PC-ions-treated Asp f 1 were 1.99 ng for sham treatment and 10.93 ng for PC-ions treatment, respectively (Fig. 3c). The result showed 81.8 % reduction of IgE-binding capacity after PC-ions treatment and this reduction was comparable with that for CFE.

## Discussion

Sensitization to cat allergens in childhood represents a strong risk factor for developing pollinosis, asthma, and atopic eczema in school children and adults [4]. To avoid sensitization to cat allergens, it is not sufficient to avoid keeping or approaching a cat, because airborne cat allergens can be carried with clothes and hairs of cat-owners into kindergartens, school class rooms, and public institutions [9]. We found that PC-ions could effectively degrade and inactivate an airborne cat allergen, Fel d 1 (Figs. 2a, c and 3a). PC-ions significantly degraded both chain 1 and chain 2 for Fel d 1 and the chain 2 disappeared almost completely after PC-ions treatment by SDS-PAGE analysis (Fig. 2c). Degradation and modification of the tertiary structure of Fel d 1 by PC-ions may also significantly lead to the impairment of its IgE-binding capacity.

PC-ions treatment of purified Asp f 1 also impaired IgE-binding capacity of Asp f 1, although exposure with PC-ions had no significant effect on the protein amount of Asp f 1 as quantified by sandwich ELISA (Fig. 3c and data not shown). Our ELISA inhibition assay showed that PC-ions treatment impaired IgE-binding capacity for CFE and purified Asp f 1, which indicated that 73.5 % and 81.8 % of their IgE-binding capacities were lost upon exposure to PC-ions (Fig. 3b and c). These degree of impaired IgE-binding capacity were comparable to that seen in PC-ions-treated Fel d 1 (76.9 %) (Fig. 3a). One possible explanation for the decreased IgE-binding capacity in purified Asp f 1 is that conformational epitopes of Asp f 1 might be destroyed by treatment with PC-ions to impair its IgE-binding capacity without affecting protein amount. Although our data suggest that induction of protein degradation is the predominant effect of PC-ions, another possibility that PC-ions induce any structural changes in purified Asp f 1 (e.g. induction of protein denaturation and/or aggregation) needs to be addressed by further analysis.

PC-ions can be generated by the discharge of plasma in a Plasma Cluster™ device. Plasma discharge can produce OH radicals from atmospheric hydrogen oxide to generate H_3_O^+^(H_2_O)_n_ positive-cluster and O_2_^−^(H_2_O)_n_ negative-cluster ions. It has been considered that charged PC-ions attach to the protein surface, hydroxyl radicals appear on it as a results of chemical interactions between the ions, and degrade it nonspecifically [17]. Neither NO_x_ nor SO_x_ are detectable during the production of PC-ions by plasma discharge (data not shown). There are several reports to show the degradation of protein and inactivation of virus by oxidation by ozone, it can be cogenerated with PC-ions by plasma discharge and other air-cleaning devices [18–20]. However, the amount of ozone cogenerated with PC-ions from our devices was negligible (less than 0.01 parts per million) and considered not to affect the protein degradation, significantly [17]. Therefore, we considered the degradation and impairment of IgE-binding capacity of the allergens were mediated by the generation of hydroxyl radicals produced by generation of PC-ions neither by generation of ozone.

We previously showed capacity of PC-ions to inactivate pollen allergens [3]. To further confirm the usefulness of PC-ions on environmental control of allergens, here we have focused on cat and fungal allergens, since those are common and clinically important indoor allergens existed throughout the year. Present data implicate that PC-ions could be useful for inactivation of indoor allergens to avoid clinical symptoms. The actual efficacy of PC-ions in normal living conditions should be confirmed by future clinical studies. In addition, the precise mechanisms whereby PC-ions degrade and/or inactivate airborne allergens needs to be further clarified.

## Conclusion

In this present study, we showed that inactivation of crude and purified airborne allergens by PC-ions in experimental cylindrical apparatus could occur. We will need to establish whether a PC-ions-generating device can degrade and inactivate airborne allergens in indoor living spaces and ameliorate allergic symptoms that can be elicited by airborne allergens in double-dummy, sham device-controlled, randomized clinical studies. Together, our data suggests that PC-ions can effectively inactivate a cat major allergen and fungal allergens by reducing the amount of allergens and impairment of their IgE-binding capacities.
